# Temporal trends in the disease burden of osteoarthritis from 1990 to 2019, and projections until 2030

**DOI:** 10.1371/journal.pone.0288561

**Published:** 2023-07-24

**Authors:** Xiaoqing Chen, Haifeng Tang, Jinding Lin, Rongdong Zeng

**Affiliations:** Quanzhou First Hospital Affiliated to Fujian Medical University, Quanzhou, Fujian Province, China; Chung Shan Medical University, TAIWAN

## Abstract

This study aimed to report trends in the global burden of osteoarthritis (OA) from 1990 to 2019 and predict the trends in the following years based on Global Burden of Diseases, Injuries, and Risk Factors Study (GBD) 2019. The study included reporting on the prevalence and incidence rates, as well as disability-adjusted life years (DALYs). Additionally, the age-standardized incidence rate (ASR) and Estimated Annual Percent Change (EAPC) were analyzed along with related factors, finally, Bayesian age-period-cohort (BAPC) analysis were utilized to predict the trends in the upcoming years. In 2019, globally, there were about 414.7 million (95%UI: 368.8 to 464.4 million) OA incident cases, with an age-standardized incidence rate (ASR) about 492.21 (95% UI:438.66 to 551.5) per 100000. And there were about 527.8 million (95% UI: 478.7 to 584.8 million) OA prevalent cases in 2019. The DALYs for OA increased to about 189.49 million (95%UI: 95.71 to 376.60 million) from 1990 to 2019 (EAPC:0.14%; 95%CI: 0.12% to 0.16%). There was a positive association between ASR and Socio-demographic index (SDI) both at the regional and national level. BAPC results showed that ASR in females would decrease but increase in males in the following years. In conclusion, the global burden of OA has risen steadily between 1990 and 2019, placing a significant strain on society. This trend is expected to continue in the coming years. To alleviate this burden, it is necessary to implement measures that target risk factors such as high body mass index.

## Introduction

Osteoarthritis (OA) is a chronic joint disease commonly found among seniors [1, 2]. Its symptoms include joint pain, stiffness, and limited movement [1, 3], while its pathological changes include cartilage degeneration, bone remodeling, bone redundancy, and joint inflammation [1, 4, 5], resulting in impaired mobility and quality of life, and some late-stage OA patients might have to turn to joint arthroplasty, which bring huge economic burden to society and physic burden to patients. Thus, the burden of OA, including incidence, prevalence and disability-adjusted life years (DALYs) need to be identified, providing basic information for policymakers about OA prevention and treatment.

In the last decade, the burden of OA was reported by some review papers [6–8]. In 214, Cross et al. [6] reported that hip and knee OA ranked as the 11th largest contributor to global disability and the 38th largest contributor in terms of disability-adjusted life years (DALYs). This highlights the significant impact of hip and knee OA on global health as a major concern. Moreover, according to Safiri et al. [7], there was a significant increase in the prevalence and incidence rate of OA between 1990 and 2017, the highest prevalence was observed in Oman, Equatorial Guinea, and the United States. Given the extended life expectancy, it is expected that the prevalence of OA will continue to rise. All these review papers indicated that the burden of OA increased in the last decades, and many measures should be taken to reduce the global burden of OA in near future. To date, no studies have examined the current global burden of OA using the latest version of the GBD study and attempted to forecast future trends.

To take a latest overview of the global burden of OA thoroughly, we showed the prevalence, incidence and DALYs of OA as reported in the GBD 2019 study, and assessed the correlation between incidence and Socio-demographic Index (SDI) at the regional and national level, finally, we predicted the trends of the burden of OA in the next 11 years by Bayesian age-period-cohort analysis.

## Materials and methods

### Overview

The Institute for Health Metrics and Evaluation (IHME) published a study titled the Burden of Diseases, Injuries, and Risk Factors 2019 (GBD 2019) [9], which is recognized as the most comprehensive and detailed analysis of diseases, injuries, and risk factors on a global scale. The latest version of the Global Burden of Disease (GBD) study, known as GBD 2019, provides estimates on the burden of 369 causes of death and disability, as well as 87 risk factors and groups of risk factors, for both males and females in 204 countries and territories worldwide [9, 10]. These estimates are provided on a global and regional level.

The GBD study utilizes a range of estimation and modeling techniques to produce comparable results on the global burden of diseases and injuries. A comprehensive account of the burden estimation process, including primary values such as incidence, prevalence, mortality, years of life lost (YLLs), years lived with disability (YLDs), and DALYs in the GBD 2019 study, has been provided in previous publications [9, 10].

### Definition of cases and sources of data

The GBD 2019 study encompassed osteoarthritis in various locations such as the hip, knee, hand, and other sites, Kellgren-Lawrence (KL) grade 2–4 detected by radiography was designated as the reference case for osteoarthritis (OA) [9]. Grade 2 osteoarthritis is indicated by the presence of visible osteophyte in the hip or knee, along with persistent pain lasting for a minimum of one month since last year. On the other hand, grade 3–4 osteoarthritis is distinguished by the presence of osteophytes, joint space narrowing, deformity, and pain that has endured for at least one month within the last 12 months [11].

IHME systematically reviewed the occurrence and frequency of OA in the population between 1980 and 2009 for GBD 2010. This review was subsequently updated for GBD 2017, which only covered hip and knee OA. However, IHME included hand and other types of OA for GBD 2019, yielding the most thorough and current data on the burden of OA available. The GHDx query tool (http://ghdx.healthdata.org/gbd-results-tool) was used to gather information on the prevalence, incidence, and DALYs of OA between 1990 and 2019. This data was sorted by gender, region, country, and etiology. In total, statistics from 204 countries and territories were included, these countries and territories were sorted into five regions based on their SDI: high, high-middle, middle, low-middle, and low. SDI is a comprehensive measure that reflects the social and economic advancement of a region by evaluating factors such as per capita income, education level, and fertility rates [12]. SDI values are graded between 0 to 1, with higher scores denoting more developed countries.

### Statistical analysis

The website provided data on the yearly occurrence of both incident and prevalent cases, as well as DALYs. The DALY measure, created by the World Bank and WHO, is becoming increasingly popular for evaluating how much the disease is impacting individual health status. It combines the years lost due to disability and the years of life lost into a single value, which is always positive and reflects the severity of healthy lifespan reduction caused by the illness. To quantify the disease burden, age-standardized rates (ASR) were calculated for incidence, prevalence, and DALYs. These rates adjust for population age differences and larger values indicate higher morbidity, mortality or DALY rates. Pearson’s correlation analysis was used to determine the relationship between SDI and ASR.

To capture the temporal trends of incidence, death, and DALYs age-standardized rates (ASR), the estimated annual percentage change (EAPC) was computed. The ASR was represented by the natural logarithm of a regression line in the form y = a+bx+c, where x is the calendar year and y is ln(ASR). The EAPC was determined as 100 * (exp[b] - 1), and a 95% confidence interval (CI) was calculated as well. If both the EAPC and the lower limit of the 95% CI are positive, the ASR is considered to be increasing. Conversely, if both the EAPC and the upper limit of the 95% CI are negative, the ASR is considered to be decreasing. To assess healthcare quality and availability in each country, the Human Development Index (HDI) was used and reported to the World Bank (http://databank.worldbank.org/data/home.aspx). Pearson’s correlation analysis was conducted to determine the association between HDI and each EAPC. Ultimately, we conducted a BAPC analysis in R with the aid of the BAPC and INLA software packages [13]. This allowed us to make projections for ASR by gender from 2019 to 2030 [14]. All data analyses were conducted using the open- source software R (version 4.2.1).

## Results

### OA burden at global level

**Fig 1:**
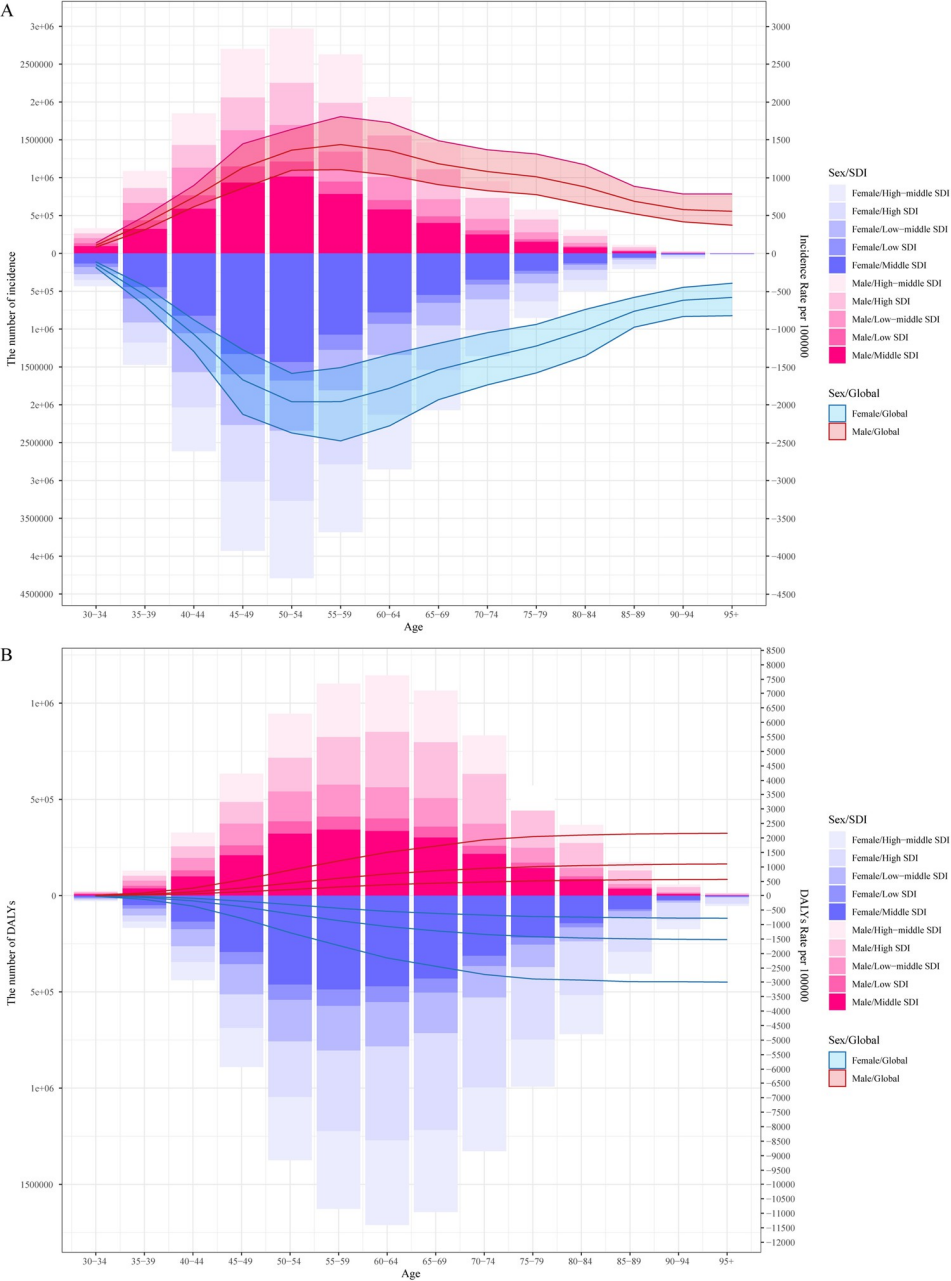
Global number and age-standardized rates of incidence (A) and DALYs (B) by sex, age and SDI regions. SDI: socio-demographic index; DALYs: disability-adjusted life years.

**Fig 2:**
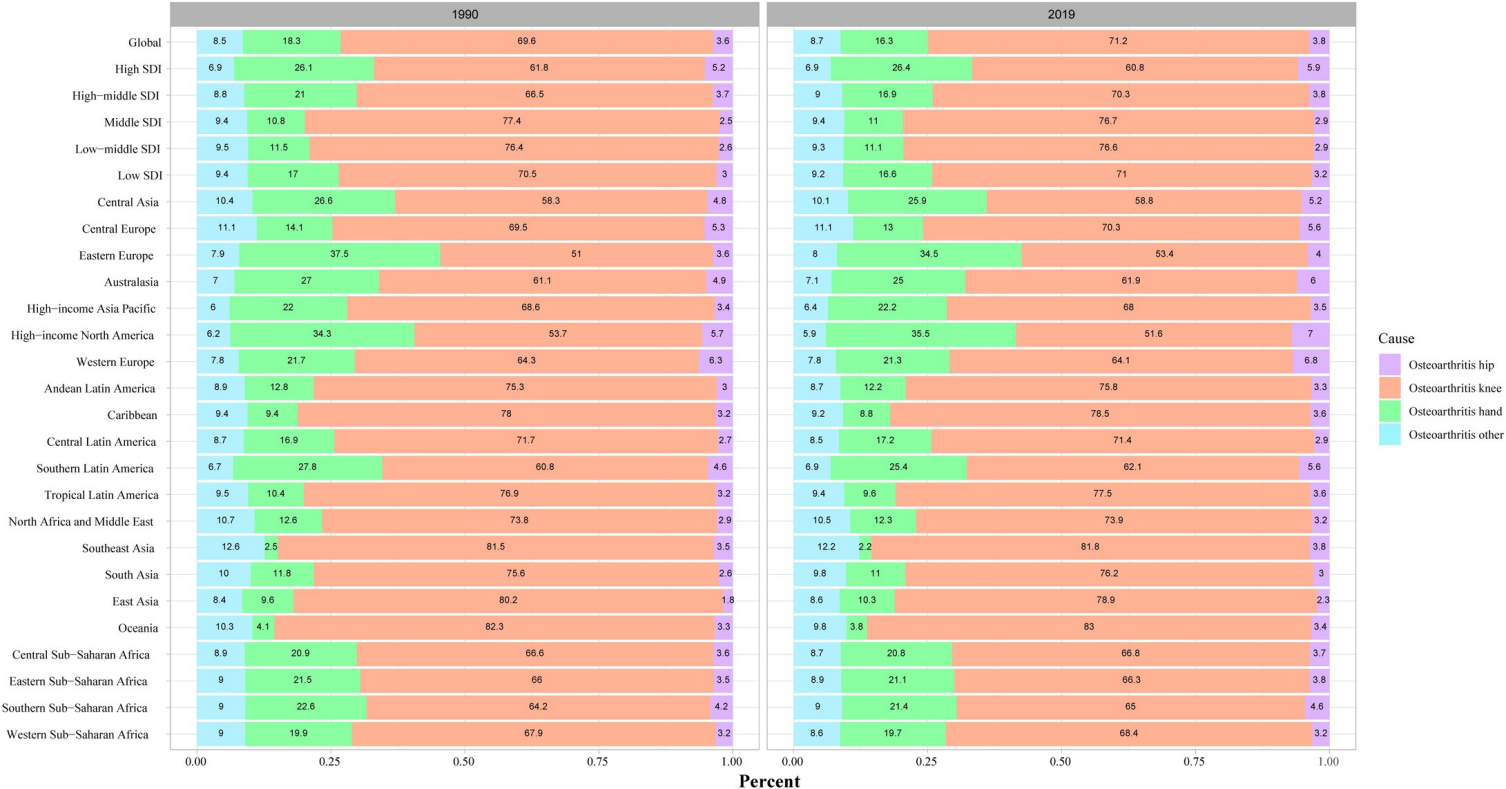
The proportion of hip, knee, hand and other sites osteoarthritis at region level, both sexes, in 1990 and 2019.

The global burden of OA was summarized in Table 1 and Fig 1. As Table 1 and Fig 1A showed, in 2019, globally, there were about 414.7 million (95%UI: 368.8 to 464.4 million) OA incident cases, with an age-standardized incidence rate (ASR) about 492.21 (95% UI:438.66 to 551.5) per 100000, the ASR was increased from 1990 to 2019 (EAPC = 0.16%; 95% CI: 0.15% to 0.18%). The largest incident cases were found in 50–54 age group both in males and females in middle SDI regions, while the highest ASR were found in 55–59 age group. There were about 527.8 million (95% UI: 478.7 to 584.8 million) OA prevalent cases in 2019, with an age-standardized prevalence rate of 6348.25 (95%UI: 5776.43 to 7023.04) per 100000, the age-standardized prevalence rate was increased from 1990 to 2019 (EAPC:0.12%; 95%CI: 0.11% to 0.14%). And the DALYs for OA also increased to about 189.49 million (95%UI: 95.71 to 376.60 million) from 1990 to 2019 (EAPC:0.14%; 95%CI: 0.12% to 0.16%), the highest DALYs were found in 60–64 age group in males and females in middles SDI regions, but, interestingly, the highest age-standardized DALYs rate were observed in males and females larger than 75 years old (Fig 1B). As shown in Fig 2, there was no evidence that a change in the percent of the hip, knee, hand and other sites OA from 1990 to 2019 (3.6%, 69.6%, 18.3%, 8.5, respectively in 1990 and 3.8%, 71.2%, 16.3%, 8.7% respectively in 2019), globally, knee OA accounts for the majority of cases, about 70% (Fig 2).

**Table 1:** The prevalence, incidence and DALYs for osteoarthritis in 2019 and its temporal trends from 1900 to 2019.

Location	Incidence			Prevalence			DALY		
	Counts (95% UI, 2019)	Age-standardized estimates (95% UI, 2019)	EAPC (95%CI, 1990–2019)	Counts (95% UI, 2019)	Age-standardized estimates (95% UI, 2019)	EAPC (95%CI, 1990–2019)	Counts (95% UI, 2019)	Age-standardized estimates (95% UI, 2019)	EAPC (95%CI, 1990–2019)
**Global**	41467542 (36875471 to 46438409)	492.21 (438.66 to 551.5)	0.16% (0.15 to 0.18)	527811871 (478667549 to 584793491)	6348.25 (5776.34 to 7023.04)	0.12% (0.11 to 0.14)	18948965 (9571298 to 37659660)	227.97 (115.31 to 452.7)	0.14% (0.12 to 0.16)
**High-middle SDI**	9868181 (8752433 to 11070906)	496.79 (441.46 to 558.36)	0.1% (0.07 to 0.13)	130175938 (117535412 to 143945318)	6366.01 (5747.55 to 7049.91)	0.01% (-0.01 to 0.04)	4676898 (2358743 to 9255864)	228.46 (115.31 to 452.39)	0.01% (-0.02 to 0.04)
**High SDI**	9477802 (8494709 to 10659394)	635.87 (568.36 to 714.76)	0.25% (0.21 to 0.28)	140966106 (128368206 to 155668140)	8180.69 (7442.48 to 9060.65)	0.22% (0.19 to 0.26)	5324252 (2710546 to 10514597)	306.74 (155.34 to 616.21)	0.28% (0.24 to 0.33)
**Low-middle SDI**	6773438 (6020315 to 7604424)	441.33 (393.4 to 494.06)	0.32% (0.3 to 0.33)	77444486 (69559489 to 86094144)	5486.73 (4950.98 to 6077.31)	0.32% (0.3 to 0.33)	2673113 (1342773 to 5328495)	189.36 (95.45 to 379.11)	0.36% (0.34 to 0.38)
**Low SDI**	2721129 (2420667 to 3082422)	423.95 (378.56 to 476.5)	0.18% (0.17 to 0.18)	29054212 (26100161 to 32632323)	5333.62 (4824.57 to 5927.69)	0.16% (0.16 to 0.17)	1000765 (507130 to 1989882)	183.97 (93.15 to 364.95)	0.19% (0.19 to 0.2)
**Middle SDI**	12607397 (11142052 to 14162635)	458.51 (406.77 to 513.7)	0.28% (0.25 to 0.32)	149931925 (134323475 to 166722251)	5748.75 (5183.89 to 6366.92)	0.3% (0.27 to 0.34)	5265521 (2635857 to 10585575)	201.78 (101.48 to 404.31)	0.34% (0.3 to 0.38)
**Andean Latin America**	294128 (263317 to 327299)	494.51 (442.66 to 550.94)	0.35% (0.34 to 0.37)	3487928 (3165190 to 3832178)	6130.76 (5571.66 to 6732.95)	0.37% (0.35 to 0.39)	123920 (62427 to 248046)	218.1 (109.96 to 433.99)	0.41% (0.39 to 0.43)
**Australasia**	259274 (230967 to 293960)	649.52 (577.71 to 733.86)	0.28% (0.24 to 0.33)	3796806 (3439151 to 4228894)	8335.23 (7537.9 to 9261.72)	0.18% (0.14 to 0.23)	143255 (72655 to 287775)	312.53 (158.11 to 631.41)	0.21% (0.16 to 0.27)
**Caribbean**	243354 (216555 to 273451)	469.58 (418.12 to 526.95)	0.25% (0.24 to 0.27)	3022624 (2727726 to 3357127)	5808.79 (5244.44 to 6439.16)	0.27% (0.26 to 0.29)	106091 (53400 to 213522)	203.91 (102.56 to 410.23)	0.29% (0.27 to 0.31)
**Central Asia**	369414 (325439 to 421162)	416.36 (371.12 to 473.06)	0.13% (0.13 to 0.14)	4290662 (3832408 to 4854632)	5518.51 (4957.3 to 6181.69)	0.1% (0.09 to 0.11)	150888 (76713 to 301366)	194.76 (98.99 to 390.02)	0.11% (0.1 to 0.12)
**Central Europe**	739828 (662314 to 831442)	412.3 (368.31 to 462.62)	0.19% (0.19 to 0.2)	10443355 (9432264 to 11547795)	5165.48 (4661.31 to 5715.94)	0.19% (0.18 to 0.19)	363687 (184150 to 718477)	179.5 (90.05 to 353.73)	0.22% (0.21 to 0.23)
**Central Latin America**	1319395 (1169767 to 1488514)	524.55 (465.92 to 590.76)	0.29% (0.28 to 0.31)	15757637 (14242793 to 17527417)	6536.84 (5908.8 to 7244.44)	0.3% (0.28 to 0.32)	560669 (285427 to 1102704)	232.87 (118.67 to 459.61)	0.35% (0.33 to 0.37)
**Central Sub-Saharan Africa**	306476 (270363 to 347083)	431.65 (383.77 to 485.57)	0.07% (0.05 to 0.08)	3201410 (2863060 to 3610691)	5580.1 (5021.93 to 6250.83)	0.07% (0.05 to 0.08)	111150 (56581 to 219365)	194.56 (99.83 to 380.99)	0.1% (0.09 to 0.11)
**East Asia**	11025646 (9684348 to 12460944)	509.13 (450.01 to 573.24)	0.36% (0.29 to 0.43)	137282805 (122010301 to 154086091)	6324.64 (5650.99 to 7078.86)	0.35% (0.28 to 0.42)	4883685 (2428307 to 9856869)	224.59 (112.43 to 451.95)	0.4% (0.32 to 0.47)
**Eastern Europe**	1802419 (1592907 to 2052448)	590.23 (523.27 to 674.83)	-0.18% (-0.29 to -0.07)	26715406 (23727276 to 30297700)	7951.4 (7060.95 to 9031.12)	-0.25% (-0.36 to -0.14)	990569 (505746 to 1997969)	293.51 (150.41 to 591.43)	-0.27% (-0.39 to -0.14)
**Eastern Sub-Saharan Africa**	927386 (821269 to 1049657)	436.66 (388.39 to 492.36)	0.16% (0.16 to 0.17)	9725547 (8720332 to 10862386)	5608.36 (5056.03 to 6260.2)	0.16% (0.15 to 0.16)	339050 (172633 to 667382)	196.3 (100.13 to 386.3)	0.2% (0.19 to 0.21)
**High-income Asia Pacific**	2088907 (1879622 to 2343286)	675.44 (601.8 to 759.94)	0.47% (0.38 to 0.56)	32215448 (29364412 to 35366664)	8369.65 (7626.92 to 9263.68)	0.48% (0.39 to 0.56)	1256647 (638659 to 2501153)	322.66 (163.41 to 640.98)	0.63% (0.51 to 0.75)
**High-income North America**	3751719 (3316055 to 4257734)	736.56 (654.21 to 833.05)	0.14% (0.01 to 0.28)	56666352 (50983534 to 63392147)	9704.65 (8749.74 to 10856.38)	0.06% (-0.06 to 0.18)	2163818 (1117826 to 4324794)	367.37 (188.71 to 739.39)	0.07% (-0.07 to 0.21)
**North Africa and Middle East**	2292214 (2035506 to 2570668)	430.41 (382.17 to 481.93)	0.28% (0.27 to 0.29)	24604611 (22080960 to 27327135)	5342.76 (4815.89 to 5907.78)	0.27% (0.26 to 0.28)	852891 (425290 to 1687138)	185.44 (92.77 to 370.2)	0.29% (0.27 to 0.3)
**Oceania**	37423 (32788 to 42193)	396.25 (351.63 to 443.16)	0.17% (0.13 to 0.21)	380453 (336954 to 427559)	4907.17 (4386.34 to 5466.45)	0.18% (0.14 to 0.22)	12939 (6383 to 26216)	166.52 (82.78 to 336.59)	0.19% (0.15 to 0.23)
**South Asia**	6661564 (5942418 to 7491336)	421.91 (375.77 to 472.43)	0.28% (0.27 to 0.29)	75631790 (68060651 to 84451436)	5219.04 (4711.22 to 5790.87)	0.32% (0.31 to 0.33)	2569765 (1297451 to 5130108)	177.2 (89.77 to 355.02)	0.36% (0.35 to 0.37)
**Southeast Asia**	2375632 (2087010 to 2662467)	336.25 (298.29 to 374.23)	0.35% (0.34 to 0.36)	26680430 (23563610 to 29924556)	4128.87 (3676.73 to 4603.34)	0.37% (0.36 to 0.38)	906248 (445552 to 1822119)	139.84 (68.94 to 281.08)	0.41% (0.4 to 0.42)
**Southern Latin America**	480229 (428683 to 543046)	617.77 (552.09 to 699.39)	0.29% (0.25 to 0.32)	6519476 (5904296 to 7276344)	8013.47 (7246.6 to 8953.5)	0.24% (0.22 to 0.27)	244276 (124500 to 487435)	299.47 (152.46 to 598.84)	0.28% (0.25 to 0.31)
**Southern Sub-Saharan Africa**	334467 (297145 to 377015)	507.3 (453.44 to 571.02)	0.18% (0.18 to 0.18)	3846179 (3463107 to 4298423)	6542.25 (5904.25 to 7279.54)	0.16% (0.16 to 0.16)	135193 (68747 to 267079)	230.53 (117.47 to 453.98)	0.16% (0.15 to 0.16)
**Tropical Latin America**	1230540 (1087889 to 1380275)	483.19 (428.62 to 541.73)	0.29% (0.28 to 0.3)	14507291 (13041412 to 16103615)	5869.83 (5280.9 to 6498.3)	0.3% (0.29 to 0.31)	508822 (255356 to 1007642)	206 (103.51 to 407.54)	0.34% (0.33 to 0.35)
**Western Europe**	3785517 (3372143 to 4249461)	556 (497.13 to 622.23)	0.25% (0.22 to 0.27)	57033769 (51909522 to 62909937)	7056.85 (6394.92 to 7801.73)	0.21% (0.19 to 0.23)	2103869 (1067688 to 4239455)	258.45 (130.74 to 514.19)	0.25% (0.23 to 0.28)
**Western Sub-Saharan Africa**	1142010 (1008100 to 1293475)	464.55 (413.06 to 522.98)	0.16% (0.12 to 0.2)	12001891 (10742999 to 13432506)	5922.65 (5346 to 6596.3)	0.14% (0.11 to 0.18)	421531 (215344 to 835156)	208.83 (106.42 to 411.09)	0.17% (0.13 to 0.21)

### OA burden at national level

**Fig 3:**
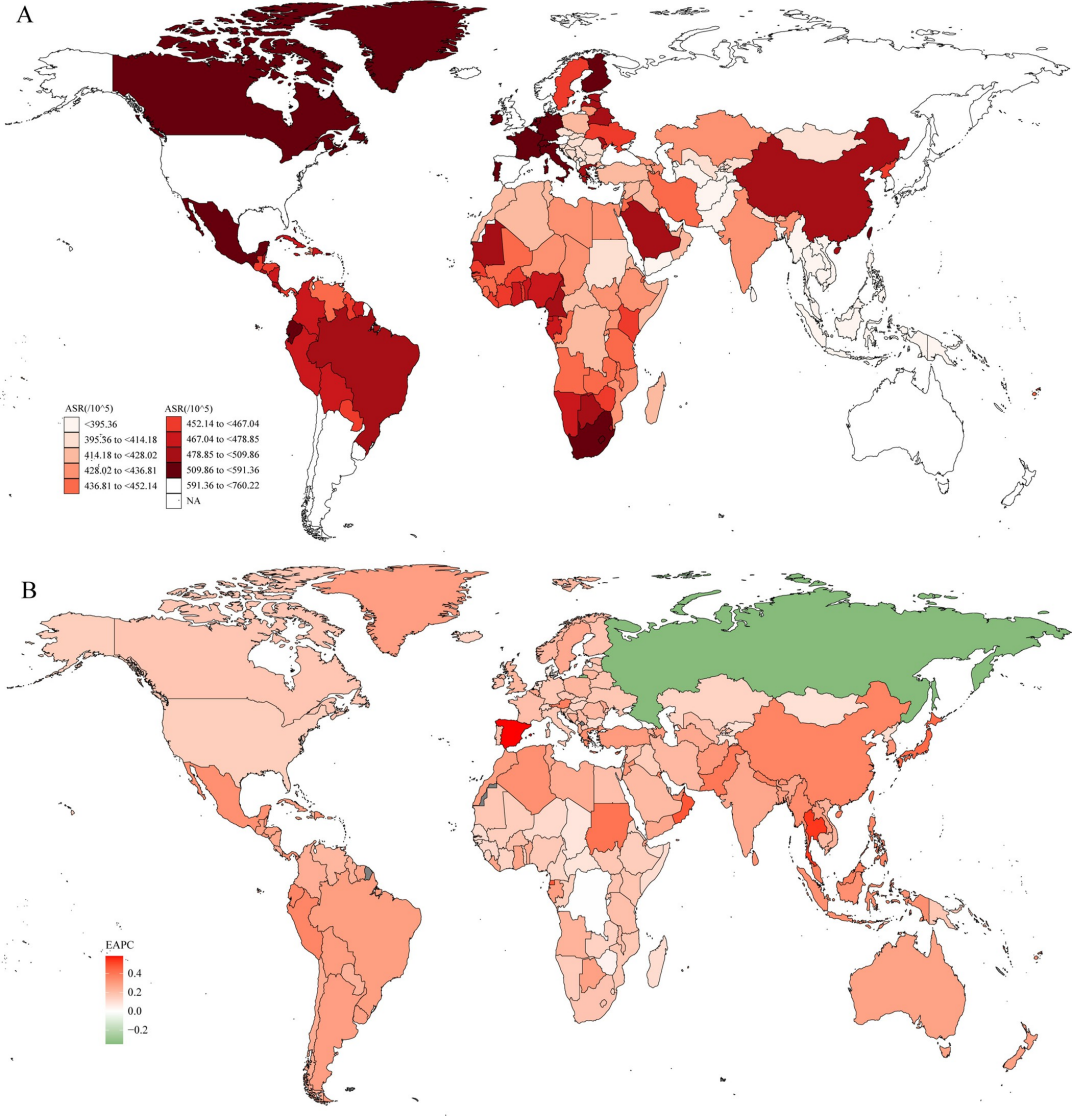
The global disease burden of osteoarthritis for both sexes in 204 countries and territories. A: the ASR of osteoarthritis in 2019; B: the EAPC of osteoarthritis in 2019. ASR: age-standardized incidence rate; EAPC: Estimated Annual Percent Change.

The ASR of OA in 2019 and the change of ASR at national level from 1990 to 2019 were summarized in Fig 3. As shown in Fig 3A, in 2019, the global ASR varied from 309.65 to 760.22 per 100000, United States of America has the highest ASR (760.22 per 100000; 95%UI: 675.98 to 859.03 per 100000), followed by the Republic of Korea (734.4 per 100000; 95%UI: 654.27 to 830.95 per 100000), Iceland (718.28 per 100000; 95%UI: 629.78 to 819.21 per 100000), Brunei Darussalam (686.3 per 100000; 95%UI: 610.76 to 775.51 per 100000), Singapore (678.69 per 100000; 95%UI: 608.33 to 766.82 per 100000).

The change of ASR at the nation level was summarized in Fig 3B. From 1990 to 2019, ASR increased at the greatest rate in Spain (EAPC:0.58%; 95%CI: 0.51% to 0.66%), followed by Thailand (EAPC:0.54%; 95%CI: 0.52% to 0.55%), Maldives (EAPC:0.49%; 95%CI: 0.46% to 0.53%), Oman (EAPC:0.48%; 95%CI: 0.46% to 0.50%), Equatorial Guinea (EAPC:0.47%; 95%CI: 0.44% to 0.50%). It is worth noting that although ASR increased in most countries, it decreased in Russian Federation (EAPC: -0.35%; 95%CI: -0.49% to -0.20%).

### The correlation between ASR and SDI

**Fig 4:**
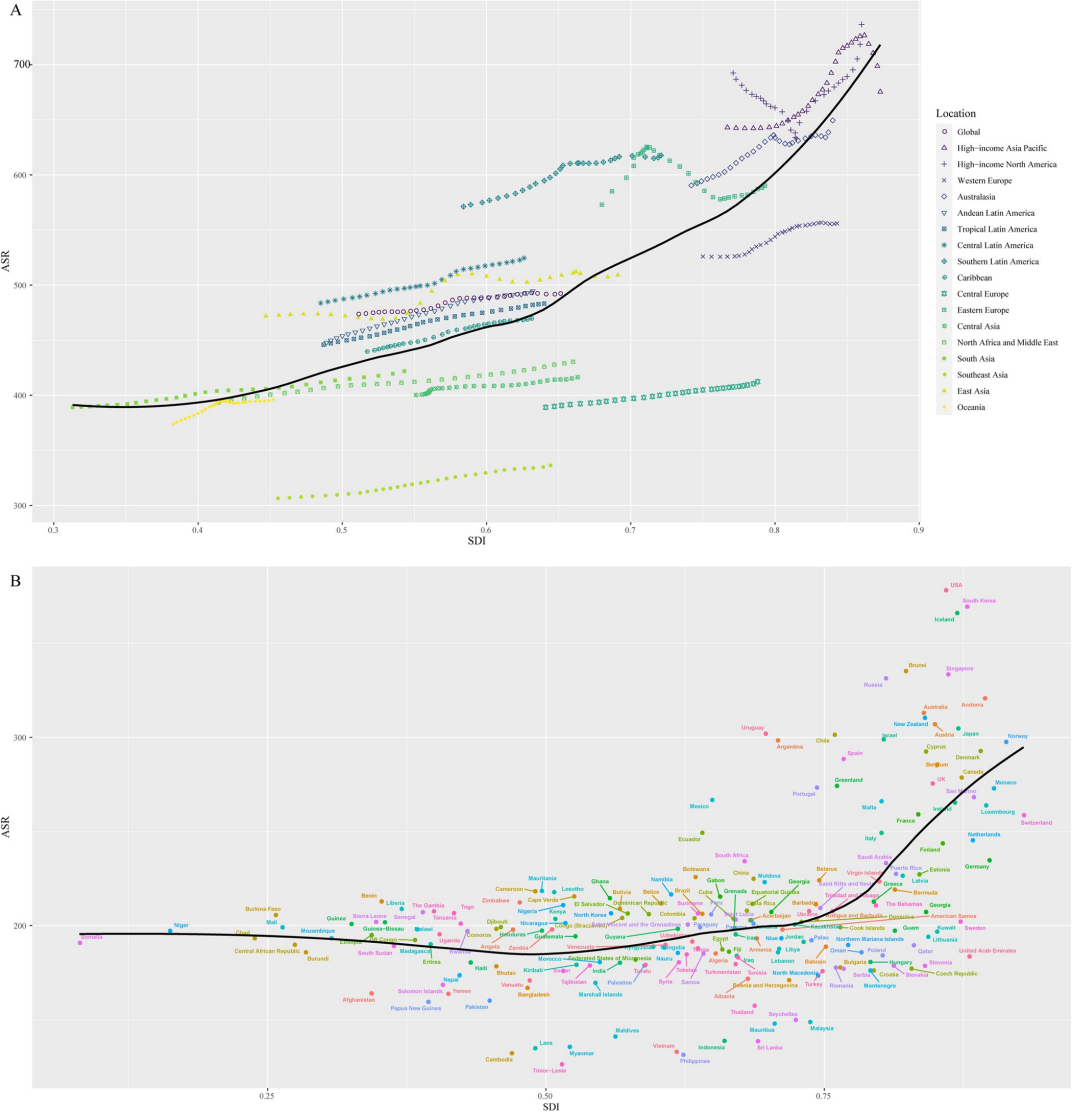
The association between ASR and SDI at the region (A) and nation (B) level. ASR: age-standardized incidence rate; Socio-demographic index (SDI).

We next explored the association between ASR and SDI. The results were summarized in Fig 4, showing that there was a significant positive association between SDI and ASR both at the region level (Fig 4A) and nation level (Fig 4B), indicating that regions or countries with a higher SDI might have a higher incidence rate of OA. As Fig 4A showed, at the region level, the ASR levels in high-income North America, Western Europe, and Southern Latin America were higher than that of the expected level based on SDI from 1990 to 2019. Fig 4B showed a positive association between SDI and ASR at the nation level, the ASR levels in some countries were higher than that of expected level based on SDI from 1990 to 2019, like Singapore, Canada, Australia and Spain.

### Analysis of EAPC’s influencing factors

**Fig 5:**
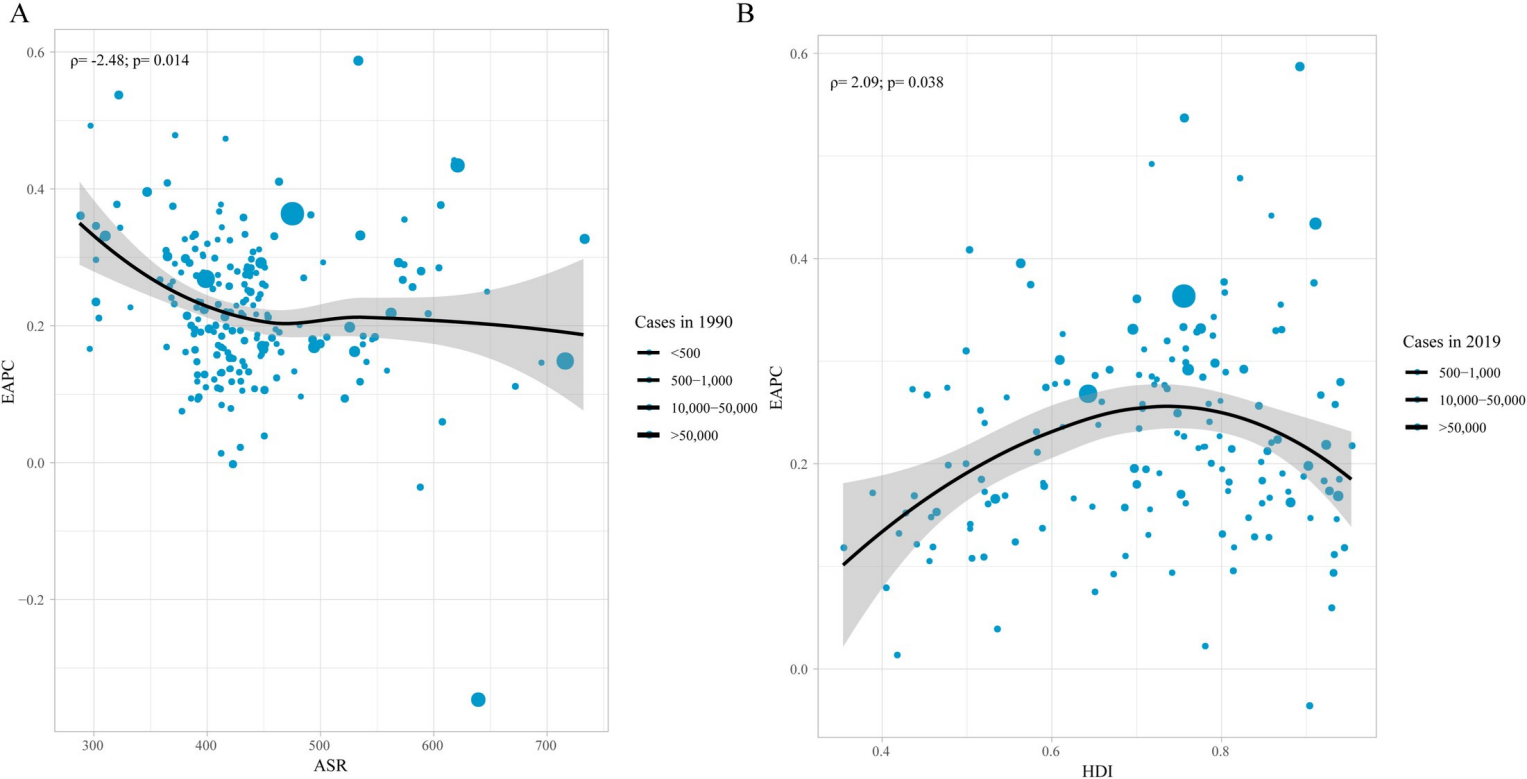
The association between EAPC and ASR (A), HDI (B). EAPC: Estimated Annual Percent Change; ASR: age-standardized incidence rate; HDI: Human development index (HDI).

We further performed an analysis on EAPC’s influencing factors, especially ASR and Human development index (HDI). The results were summarized in Fig 5, there was significant correlation between EAPC and ASR in 1990, HDI in 2019. Specifically, as Fig 5A showed, there was a negative correlation between ASR and EAPC (ρ = -2.48; *P* = 0.014) when ASR was less than 500 per 100000, but this correlation disappeared when ASR was large than 500 per 100000; and as Fig 5B showed, when HDI was limited to below 0.7, there was a positive correlation between HDI and ASR (ρ = 2.09; *P* = 0.038), indicating that those countries with higher HDI might have a more rapid increase in ASR.

### Risk factors attributable to OA burden

**Fig 6:**
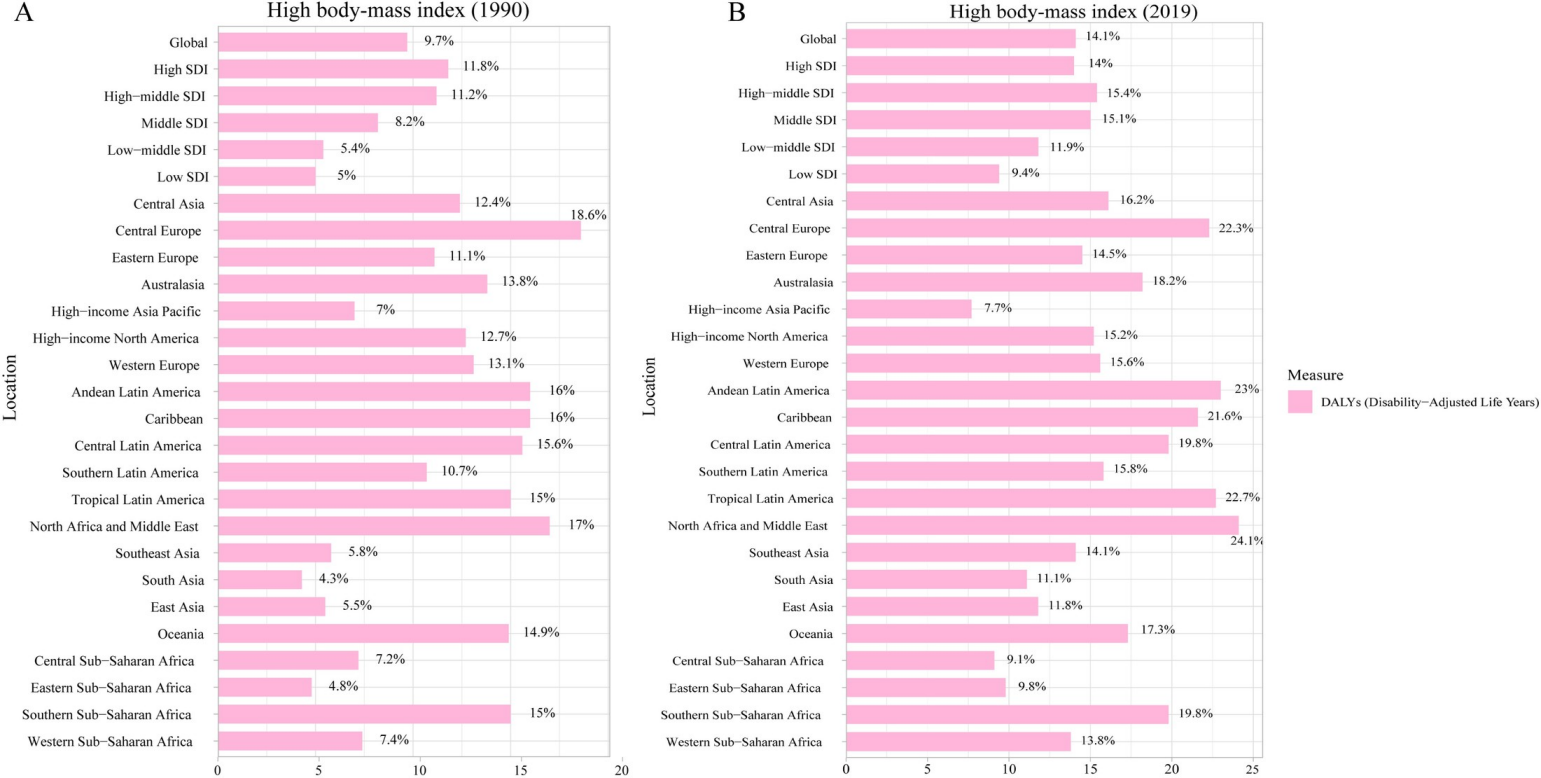
The risk factor of osteoarthritis in 1990 (A) and 2019 (B).

To further identified risk factors of OA, we obtained all risk factors of OA from GBD 2019. The results were summarized in Fig 6. The results showed that metabolic risks were the main risk factors for OA, specifically, high body-mass index. As shown in Fig 6A, globally, the percent of DALYs attributed to high body-mass index was 9.7% in 1990, and 11.8%, 11.2%, 8.2%, 5.4%, 5% in high SDI, high-middle SDI, middle SDI, low-middle SDI and low SDI regions, respectively. By 2019, the percent of DALYs attributed to high bode-mass index increased to 14.1% globally, and 14%, 15.4%, 15.1%,11.9%, 9.4% in in high SDI, high-middle SDI, middle SDI, low-middle SDI and low SDI regions, respectively (Fig 6B). In Western and Central Europe, North Africa and the Middle East, and Tropical Latin America, high body-mass index was the major risk of OA.

### Projection of OA

**Fig 7:**
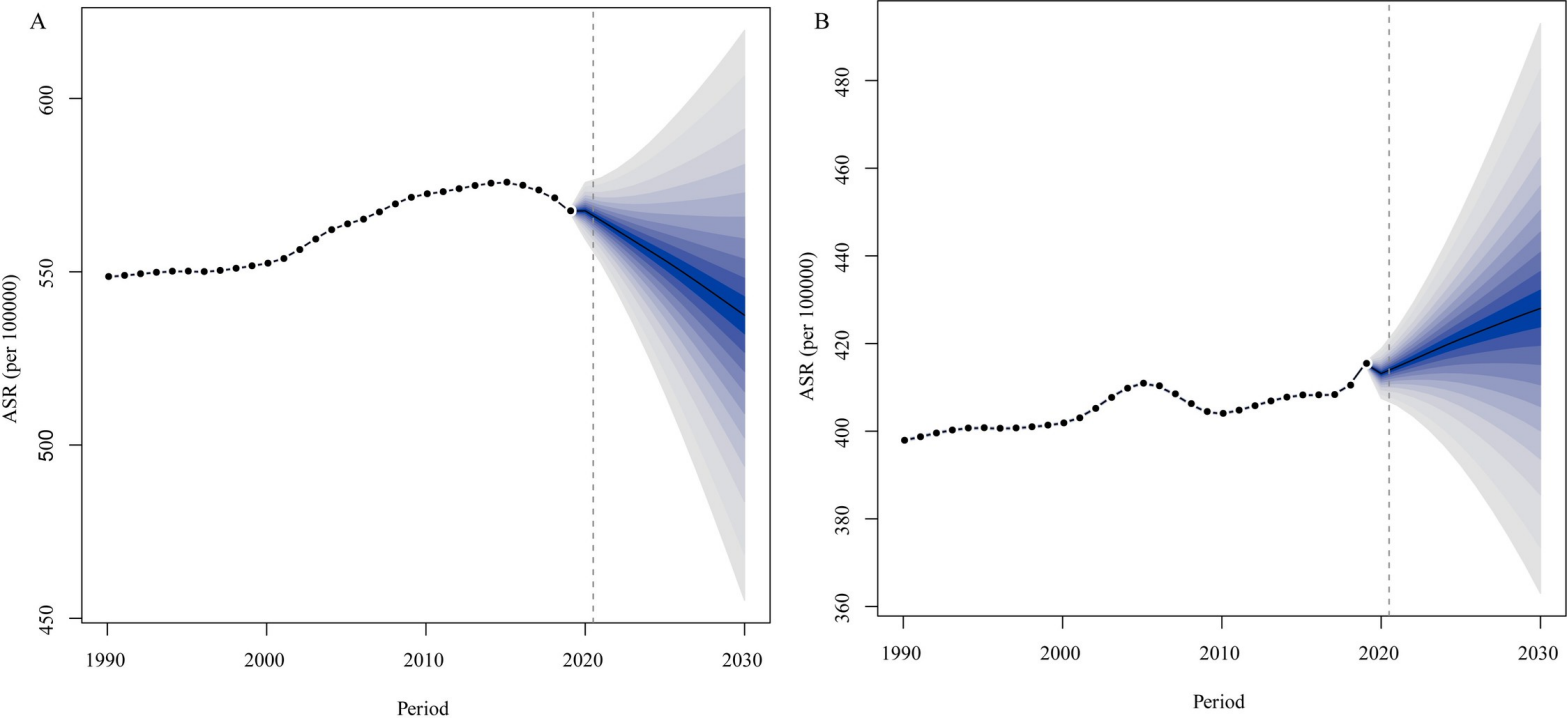
Trends of ASR from 2019 to 2030 in females (A) and males (B) predicted by Bayesian age-period-cohort (BAPC) models. ASR: age-standardized incidence rate.

To learn about the trends of ASR of OA after 2019, we used Bayesian age-period-cohort models to predict the ASR from 2019 to 2030 by sex, the results were summarized in Fig 7. As Fig 7A showed, the ASR in females would decrease annually after 2019 from 567.62 per 100000 in 2019 to 537.45 per 100000 in 2030. However, the predictive results showed that ASR in males would increase annually (Fig 7B), from 415.40 per 100000 in 2019 to 428.00 per 100000 in 2030. These results indicated that the trends of ASR in females would decrease but increase in males.

## Discussion

Osteoarthritis (OA) is one of the leading causes of disability in the world, it has been reported that the incidence rate increased year by year [4, 15], which bring a huge burden on the health care system due to its high disability rate. Although many papers have reported the global burden of OA, these papers were based on GBD 2010 study and GBD 2017 study [6, 7], in which only hip and knee OA were included. In this study, we presented the most comprehensive and up-to-date global burden of OA, and provided the trends of OA incidence in the following years.

With the continuous development of medical diagnosis and treatment technology, there has been significant improvement in the diagnostic level of osteoarthritis. On the one hand, the advancement of imaging technology has made the diagnosis of osteoarthritis more accurate. For example, imaging techniques such as X-rays, magnetic resonance imaging (MRI), and computed tomography (CT) can accurately locate and evaluate the extent, location, and type of osteoarthritis lesions, which is very important for formulating treatment plans. On the other hand, the use of biomarkers and gene detection technology can also help diagnose osteoarthritis. For example, indicators such as C-reactive protein (CRP) and lactate dehydrogenase (LDH) can be used as auxiliary diagnostic markers for osteoarthritis; gene sequencing can identify genetic factors related to osteoarthritis, thus achieving early prevention and intervention. Overall, with the continuous development of medical diagnosis and treatment technology, the diagnostic level of osteoarthritis has also significantly improved.

The prevalence, incidence and DALYs of OA kept increasing from 1990 to 2019, which were reported in previous papers. The prevalence, incidence and DALYs of OA kept increasing from 1990 to 2019, which were reported in previous papers [6, 7]. By 2019, there were about 414.7 million OA incident cases, with an age-standardized incidence rate (ASR) of about 492.21 per 100000, and about 527.8 million OA prevalent cases in 2019, the DALYs increased to about 189.49 million. At national level, the United States of America has the highest ASR, followed by the Republic of Korea, Iceland, Brunei Darussalam, and Singapore. As for the increasement of ASR, Spain has the most rapid increase rate from 1900 to 2019, followed by Thailand, Maldives, Oman, and Equatorial Guinea.

We further analyzed the ASR- and EAPC-related factors. A positive association existed between SDI and ASR both at the region and nation level, indicating that with a higher SDI, there might be a higher ASR, which was similar to report conducted by Safiri et al. [7]. Besides, we found that there was a negative association between ASR and EAPC while a positive association between HDI and EAPC to some extent, indicating that in some high-income countries, the ASR might increase more rapidly, this has not been reported before.

The global burden of OA increased annually, and there are no sure remedies for OA, although many late-stage OA patients might receive joint arthroplasty, complications including periprosthetic joint infection, aseptic loosening and dislocation [16, 17], etc., might occur. So it is important to identify any potential risk factors of OA, which would be helpful for policymakers to take measures for OA prevention. We analyzed all risk factors of OA included in the GBD 2019 study, and found that metabolic risks, specifically, high bode-mass index, was the only risk factors for OA. And the percentage of DALYs due to OA attributed to high body-mass index significantly increased from 1990 to 2019. It has been reported that the number of overweight and obese people increased rapidly in the last decades [18–20], and has become a global health issue. Thus, as for policymakers, many measures targeting high body-mass index should be taken to reduce the risk of OA, as well as other obesity-related diseases [20–23], especially in middle SDI, high-middle SDI and high SDI regions.

The trends of OA in the following years predicted by BAPC in this study indicated that the ASR in females would decrease from 2019, while increasing in males. A previous study showed that the global burden of OA was higher in females, so many prevention and treatment strategies should be taken in this group, however, based on BAPC predictive results, the global burden of OA in males would increase, thus much more attention should also be paid in this group, especially in prevention.

As described in previous studies [6, 7, 23–25], there were some limitations in this study:1) this study was based on GBD 2019 study, since OA measurement is heterogeneous, it is difficult to compare estimates across populations and can lead to serious errors; 2) for OA cases, the quality of life is very important, but this index hasn’t included in GBD 2019 study, further work about the quality of life should be done and analyzed.

## Conclusions

In conclusion, the present study showed the most comprehensive and up-to-date picture of the global burden of OA. The global burden of OA increased significantly from 1990 to 2019, and is going to increase in the following years, high body-mass index is the risk factor for OA, many measures targeting high body-mass index should be taken to prevent the incidence of OA.
