# Correction: Temporal trends in the disease burden of osteoarthritis from 1990 to 2019, and projections until 2030

**DOI:** 10.1371/journal.pone.0333919

**Published:** 2025-10-06

**Authors:** Xiaoqing Chen, Haifeng Tang, Jinding Lin, Rongdong Zeng

In the Abstract section, there is an error in the fourth and sixth sentence. The correct sentence are: In 2019, globally, there were about 41.47 million (95%UI: 368.8 to 464.4 million) OA incident cases, with an age-standardized incidence rate (ASR) about 492.21 (95% UI: 438.66 to 551.5) per 100000. And there were about 527.8 million (95% UI: 478.7 to 584.8 million) OA prevalent cases in 2019. The DALYs for OA increased to about 18.949 million (95%UI: 95.71 to 376.60 million) from 1990 to 2019 (EAPC:0.14%; 95%CI: 0.12% to 0.16%).
